# Characterization and phylogenetic consideration of the complete chloroplast genome of *spiraea uratensis* (rosaceae)

**DOI:** 10.1080/23802359.2025.2544686

**Published:** 2025-08-07

**Authors:** Yan Wang, Yiluo Wang, Erdong Zhang, Lei Zhang

**Affiliations:** Key Laboratory of Ecological Protection of Agro-pastoral Ecotones in the Yellow River Basin National Ethnic Affairs Commission of the People’s Republic of China, School of Biological Science & Engineering, North Minzu University, Yinchuan, P. R. China

**Keywords:** *Spirea uratensis*, rosaceae, chloroplast genome, phylogenetic analysis

## Abstract

*Spirea uratensis* is a shrub plant valued for its ecological and ornamental qualities. In this study, we assembled the complete chloroplast genomes of *S. uratensis*. The result revealed a 155,876 bp circular structure with typical quadripartite organization: 84,291 bp LSC, 18,893 bp SSC, and two 26,346 bp IRs. The genome contains 132 genes and has 36.8% GC content. Phylogenetic analyses resolved *S. uratensis*, *S. henryi*, *S. ovalis*, *S. nipponica*, and *S. trichocarpa* forming a monophyletic group, confirming their close relationship. This study advances understanding of chloroplast evolution in *Spirea* of Rosaceae.

## 1. Introduction

Chloroplasts are semi-autonomous replicating organelles in plant cells and play a significant role in carrying out photosynthesis, as they convert light energy into chemical energy for storage (Kan et al. 2024). Therefore, chloroplasts are critically important not only for plants but also for humans (Yan et al. 2024). In most plant species, chloroplast genomes exhibit uniparental inheritance (predominantly maternal), with characteristics such as compact size, haploid nature, and rapid fixation time, which make it particularly valuable for analyzing nucleotide diversity and reconstructing phylogenetic relationships among closely related species (Xie et al. 2022; Zhang et al. 2024; Yisilam et al. 2025). With the rapid development of high-throughput sequencing technology, data on chloroplast genomes have seen explosive growth (Zhang et al. 2017, 2024; Jia et al. 2024). In recent years, comparative and phylogenetic analyses of chloroplast genomes have proven to be an ideal tool for species identification, resolution of phylogenetic relationships, and reconstruction of evolutionary histories (Wang et al. 2023; Quan et al. 2024; Xue et al. 2024).

*Spirea uratensis* Franchet 1883 is a shrub plant belonging to the *Spirea* (Franchet 1883) of subfamily Amygdaloideae (Rosaceae), mainly distributed in Shaanxi, Gansu, Ningxia and Inner Mongolia of Northwest China (Lu and Alexander 2003; Poliakova et al. 2022). It thrives in slopes and in valleys at an altitude of 900 to 1,100 meters and plays a crucial ecological role in the local phytocoenosium (Poliakova et al. 2022). *Spirea* has beautiful flowers, is strong and vigorous, and has the advantages of cold and drought resistance. It is an important ornamental horticultural plant (Laczkó et al. 2024). In recent years, some developments have been made in the study of chloroplast genomes of *Spirea* plants. At present, the chloroplast genomic data of most species of *Spirea* plants have been made public (Zhang et al. 2023). However, the chloroplast genomic characteristics of *Spirea* and its evolutionary relationship with other plants have not yet been studied.

In this study, we assembled and analyzed the complete chloroplast genome of *S. uratensis* for the first time. Our aims of this study were (1) to elucidates characterize the structural features of the chloroplast genome for the *S. uratensis*, and (2) to resolve the evolutionary relationships of *S. uratensis*, and to provide data support for the species identification and phylogenetic relationship of *Spirea*.

## 2. Materials

**Figure 1.**
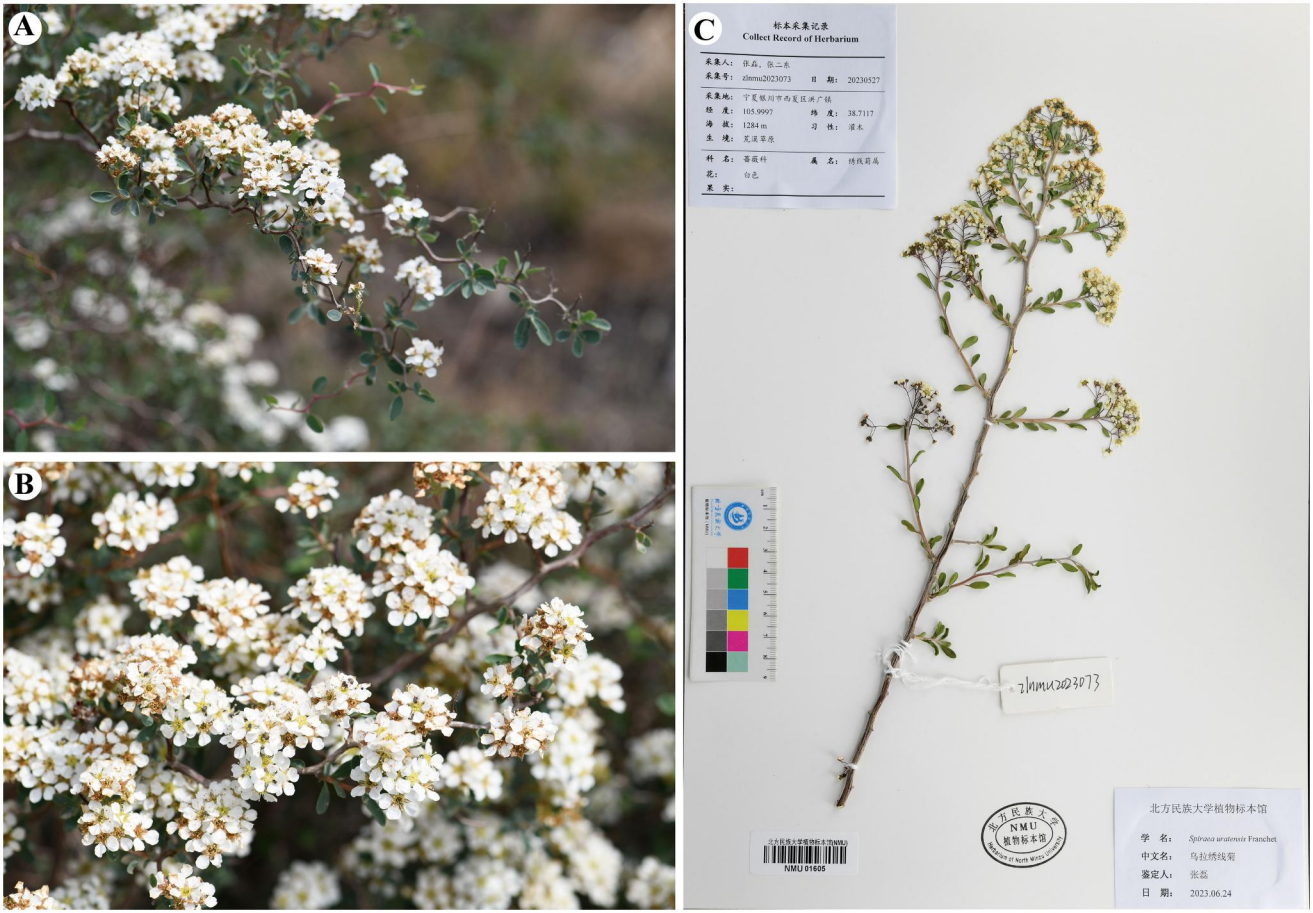
Field photos of *S. uratensis* (photographed by Dr. Lei Zhang). (A) Flower branches (branchlets terete; leaf margin entire, glabrous on both surfaces); (B) inflorescences (corymbs compound, rachis and pedicels glabrous); (C) Herbarium specimen of *S. uratensis.*

Healthy fresh leaves of *S. uratensis* were collected from Hongguang town in Xixia district (Yinchuan, Ningxia, China; coordinates: 105.9997 E, 38.7117 N; altitude: 1284 m) by Lei Zhang (zhangsanshi-0319@163.com), and subsequently desiccated using silica gel for DNA extraction. A specimen was deposited at Herbarium of North Minzu University (https://www.cvh.ac.cn/ins/info.php?code=NMU, Lei Zhang: zhangsanshi-0319@163.com) under the voucher number zlnmu2023073 (Figure 1).

## 3. Methods

Total genomic DNA was isolated using a modified CTAB method (Doyle and Doyle 1987). The NEBNext DNA Library Kit was employed to construct sequencing libraries following the manufacturer’s instructions. DNA was randomly fragmented to a size of 350 bp, and the library was sequenced on the Illumina NovaSeq 6000 platform with 150 bp paired-end reads. We obtained 6.2 Gb of high-quality paired-end reads for *S. uratensis*. After adapter trimming, the chloroplast genome was assembled using NOVOPlasty v4.3.3 (Dierckxsens et al. 2017), with the complete chloroplast genome sequence of *Spirea chinensis* (OR513055) serving as a reference. The assembled chloroplast genome was annotated using Plann v1.1 (Huang and Cronk 2015), and manual corrections were performed in Geneious v11.0.3 (Kearse et al. 2012). Sequencing depth coverage was assessed using Samtools (Danecek et al. 2021). To further clarify the phylogenetic position of *S. uratensis* in *Spirea*, we retrieved the chloroplast genomes of 28 representative species from NCBI GenBank and reconstructed phylogenetic trees using *Physocarpus amurensis* as the outgroup. All sequences were aligned using MAFFT v7.313 (Katoh et al. 2019). Maximum Likelihood (ML) analysis was performed using RAxML v8.1.24 (Stamatakis 2014) under the GTR + Γ model. The optimal model (GTR+I + G) was identified using jModeltest and Bayesian inference (BI) analysis was conducted in MrBayes v 3.2.6 (Ronquist et al. 2012). The resulting phylogenetic trees were visualized using FigTree v1.4.4 (Rambaut 2018).

## 4. Results

**Figure 2.**
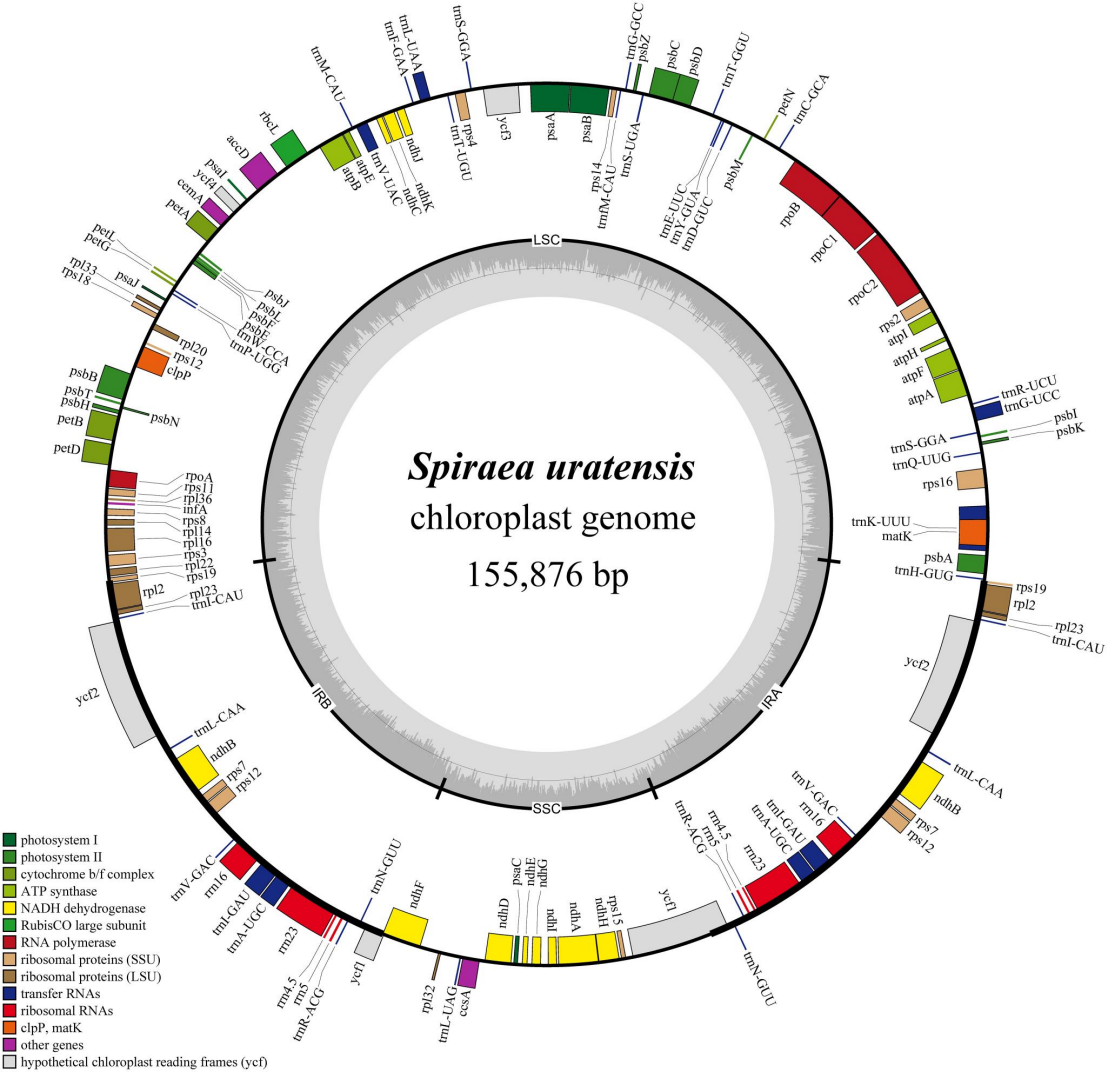
The detailed genome map of *S. uratensis* cp genome. Gene models including protein-coding genes, tRNA genes and rRNA genes are shown with various colored boxes in the outer track. The bold lines of the inner circle outline the extent of the inverted repeat regions (IRA and IRB), dividing the genome into small single-copy (SSC)and large single-copy (LSC) regions. Genes located on the inner and outer parts of the outer circle are transcribed in a clockwise and counterclockwise direction, respectively. GC content (light gray) are shown in the inside track.

**Figure 3.**
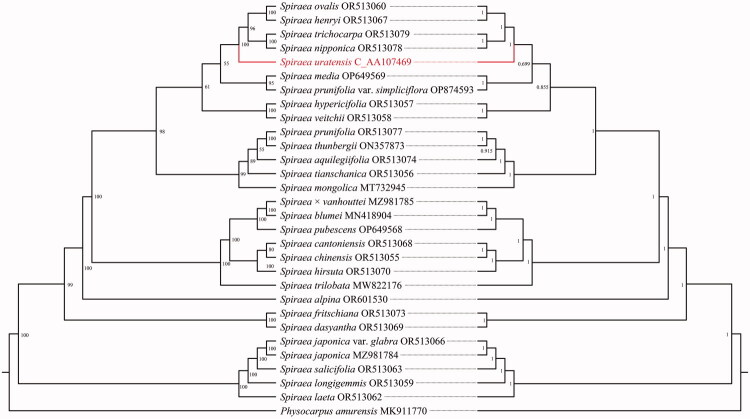
Phylogenetic tree based on whole chloroplast genome sequences for *spiraea* species with *physocarpus amurensis* as the outgroup. The left and right trees are use the maximum likelihood (ML) and bayesian inference (BI) methods respectively. GenBank accession numbers: *Spirea japonica* MZ981784 (Wang et al. 2022), *spiraea mongolica* MT732945 (Ma et al. 2021), *spiraea media* OP649569 (Song et al. 2024), *spiraea pubescens* OP649568 (Song et al. 2024), *spiraea thunbergii* ON357873 (Shen et al. 2022), *spiraea blumei* MN418904 (Huo et al. 2019), *spiraea trilobata* MW822176 (Qin et al. 2022), *spiraea prunifolia* var. *simpliciflora* OP874593 (Jeongjin et al. 2023), *spiraea chinensis* OR513055 (Zhang et al. 2024), *spiraea trichocarpa* OR513079 (Zhang et al. 2024), *spiraea nipponica* OR513078 (Zhang et al. 2024), *spiraea prunifolia* OR513077 (Zhang et al. 2024), *spiraea aquilegiifolia* OR513074 (Zhang et al. 2024), *spiraea fritschiana* OR513073 (Zhang et al. 2024), *spiraea hirsuta* OR513070 (Zhang et al. 2024), *spiraea dasyantha* OR513069 (Zhang et al. 2024), *spiraea cantoniensis* OR513068 (Zhang et al. 2024), *spiraea henryi* OR513067 (Zhang et al. 2024), *spiraea japonica* var. *Glabra* OR513066 (Zhang et al. 2024), *spiraea salicifolia* OR513063 (Zhang et al. 2024), *spiraea laeta* OR513062 (Zhang et al. 2024), *spiraea ovalis* OR513060 (Zhang et al. 2024), *spiraea longigemmis* OR513059 (Zhang et al. 2024), *spiraea veitchii* OR513058 (Zhang et al. 2024), *spiraea hypericifolia* OR513057 (Zhang et al. 2024), *spiraea tianschanica* OR513056 (Zhang et al. 2024), *spiraea* x *vanhouttei* MZ981785 (Chen et al. 2022), *spiraea alpina* OR601530, *physocarpus amurensis* MK911770.

After quality control and preprocessing, we obtained at least 4 gigabases (Gb) of whole-genome sequencing data. The clean reads were then used for reference-guided assembly, yielding a high-quality chloroplast genome. The total chloroplast genome of *S. uratensis* was 155,876 bp in length and depth for average, maximal and minimal were 4630.97x, 6284x and 442x (Supplementary Figure S1). It exhibited a typical quadripartite structure, which consisted of a pair of inverted repeat (IR) regions of 26,346 bp each, a small single-copy (SSC) region of 18,893 bp, and a large single-copy (LSC) region of 84,291 bp (Figure 2). The GC content of the *S. uratensis* chloroplast genome was 36.8%, with corresponding values of 34.62% in the LSC, 30.3% in the SSC, and 42.49% in the IR regions. In addition, a total of 132 genes were annotated in the chloroplast genome, including 84 protein-coding genes, 8 ribosomal RNA genes (rRNAs), and 37 transfer RNA genes (tRNAs). In the genome, *rps*12 and *ycf*3 contain two introns. Meanwhile, 10 protein-coding genes (*rps*16, *atp*F, *rpo*C1, *clp*P, *pet*B, *pet*D, *rpl*16, *rpl*2, *ndh*B, *ndh*A) and 6 transfer RNA genes (*trn*K-UUU, *trn*G-UCC, *trn*L-UAA, *trn*V-UAC, *trn*I-GAU, *trn*A-UGC) contain one introns (Figure 2; Table 1). The chloroplast genome contained 1 trans-splicing gene (*rps*12; Supplementary Figure S2) and 11 cis-splicing genes (*rps*16, *atp*F, *rpo*C1, *ycf*3, *clp*P, *pet*B, *pet*D, *rpl*16, *rpl*2, *ndh*A, and *ndh*B; Supplementary Figure S3). We performed phylogenetic analyses of complete chloroplast genomes from 30 *Spirea* species using Maximum Likelihood (ML) and Bayesian inference (BI). The resulting trees (Figure 3) showed congruent topologies with strong nodal support, consistently placing *S. uratensis* in a well-supported clade with four congeneric species: *S. henryi*, *S. ovalis*, *S. nipponica*, and *S. trichocarpa*. This robust phylogenetic reconstruction confirms the taxonomic position of *S. uratensis* in *Spirea* of Rosaceae.

**Table 1.** Functional classifcation of chloroplast genes of *spiraea uratensis.*

Classifcation	Gene family	Gene name
Photosynthesis related genes	Subunits of photosystem I(5)	*psa*A, B, C, I, J
	Subunits of photosystem II(15)	*psb*A, B, C, D, E, F, H, I, J, K, L, M, N, T, Z
	Subunits of ATP synthase(6)	*psb*A, B, E, F*, H, I
	Subunits of NADH-dehydrogenase(12)	*ndh*A*, B*(x2), C, D, E, F, G, H, I, J, K
	Subunits of cytochrome b/f complex(6)	*pet*A, B*, D*, G, L, N
	Subunit of rubisco(1)	*rbc*L
Transcription and translationrelated genes	Large subunit of ribosome(11)	*rpl*14, 16*, 2*(x2), 20, 22, 23(x2), 32, 33, 36
	Small subunit of ribosome(15)	*rps*11, 12**(x2), 14, 15, 16*, 18, 19(x2), 2, 3, 4, 7(x2), 8
	DNA dependent RNA polymerase(4)	*rpo*A, B, C1*, C2
	RNA Ribosomal RNAs(8)	*rrn*16(x2), 23(x2), 4.5(x2), 5(x2)
	RNATransfer RNAs(37)	*trn*A-UGC*(x2), C-GCA, D-GUC, E-UUC, F-GAA, *f*M-CAU, G-GCC, G-UCC*, H-GUG, I-CAU(x2), I-GAU*(x2), K-UUU*, L-CAA(x2), L-UAA*, L-UAG, M-CAU, N-GUU(x2), P-UGG, Q-UUG, R-ACG(x2), R-UCU, S-GGA(x2), S-UGA, T-GGU, T-UGU, V-GAC(x2), V-UAC*, W-CCA, Y-GUA
Others genes	c-type cytochrom synthesis gene(1)	*ccs*A
	Envelop membrane protein(1)	*cem*A
	Maturase(1)	*mat*K
	Protease(1)	*clp*P*
	Subunit of Acetyl-CoA-carboxylase(1)	*acc*D
	Translational initiation factor(1)	*inf*A
Unknown function genes	Conserved open reading frames(6)	*ycf*1(x2), 2(x2), 3**, 4

#*represents a gene with one intron, **represents a gene with two introns, (x2) indicates two copies.

## 5. Discussion and conclusion

In this study, we characterized the complete cp genomes of *S. uratensis*. The results demonstrated that *S. uratensis* comprised two copies of IR region, one SSC region and one LSC region (Figure 2), Which is similar to the classical quadripartite structure of previously reported *Spirea* species (Zhang et al. 2023). Previous research has shown that the chloroplast genomes of angiosperms typically range in length from 120 to 180 kb, with the inverted repeat (IR) region spanning approximately 20 to 30 kb (Wolf et al. 2011). In this study, the complete cp genome of *S. uratensis* was assembled with a total sequence length of 155,876 bp, and the length of the IR region was 26,346 bp. This further indicates the conservation of the *S. uratensis* cp genome. The average GC content of the cp genome of the *S. uratensis* was 36.8%, which was consistent with the Rosaceae plants was 35%-40% (Zhang et al. 2023), indicating the conserved genomic evolution of the *S. uratensis* (Zhang et al. 2017). The GC content in the IR region is higher than that in the LSC and SSC regions, which is also consistent with the characteristics of the chloroplast genomes of other Rosaceae plants (Zhang et al. 2023).

Some *Spirea* species have relatively similar morphological characteristics and complex genetic relationships (Drábková et al. 2017). For these closely related species, DNA fragments cannot be effectively distinguished. Therefore, it is necessary to conduct comparative studies using the chloroplast genome (Khan et al. 2018). Compared with DNA fragments, the cp genome have shown substantial power in solving phylogenetic relationships among angiosperms (Yang et al. 2022; Zhang et al. 2022). In this study, a phylogenetic tree was developed utilizing the BI method and the ML method. The tree revealed that the cp genomes of Rosaceae species dividing into four main clades with strong support, with *S. uratensis*, *henryi*, *S. ovalis*, *S. nipponica*, *and S. trichocarpa* formed a monophyletic group. This phylogenetic result was consistent with Yu et al. (2018) and Zhang et al. (2023). By analyzing the sequence and structural information of the chloroplast genomes of *S. uratensis*, we have determined their genetic evolutionary position and relationships with other *Spirea* species. This information establishes a foundation for genetic diversity, and phylogenetics of *Spirea* species. Furthermore, our study has signifcantly contributed to the enrichment of the chloroplast genome database for *Spirea* plants. Further investigation at the population level and indepth genome analysis is necessary to thoroughly understand the structural variation of the chloroplast genome in *S. uratensis*.

## Supplementary Material

Supplementary File.docx

## Data Availability

The sequenced data supporting the findings of this study are openly available in the Genome Sequence Archive in National Genomics Data Center, China National Center for Bioinformation under the accession no. C_AA107469. The associated BioProject, CRA and Bio-Sample numbers are PRJCA039036, CRA024919 and SAMC5008197, respectively.
