# Supplementary material for: Characterization and phylogenetic consideration of the complete chloroplast genome of spiraea uratensis (rosaceae)
Source: Mitochondrial DNA B Resour. 2025 Aug 7;10(9):789–94. doi: 10.1080/23802359.2025.2544686 (PMC12333033; doi:10.1080/23802359.2025.2544686)
Supplement: Supplementary File.docx [file TMDN_A_2544686_SM3949.docx]

Characterization and phylogenetic consideration of the complete chloroplast genome of *Spiraea uratensis* (Rosaceae)

Yan Wang^1^, Yiluo Wang^1^, Erdong Zhang^1^, Lei Zhang^1*^

^1^Key Laboratory of Ecological Protection of Agro-pastoral Ecotones in the Yellow River Basin National Ethnic Affairs Commission of the People’s Republic of China, School of Biological Science & Engineering, North Minzu University, Yinchuan, Ningxia, P. R. China

***Corresponding author**: Lei Zhang, zhangsanshi-0319@163.com

**ORCID**: Lei Zhang; https://orcid.org/0000-0001-5301-4658


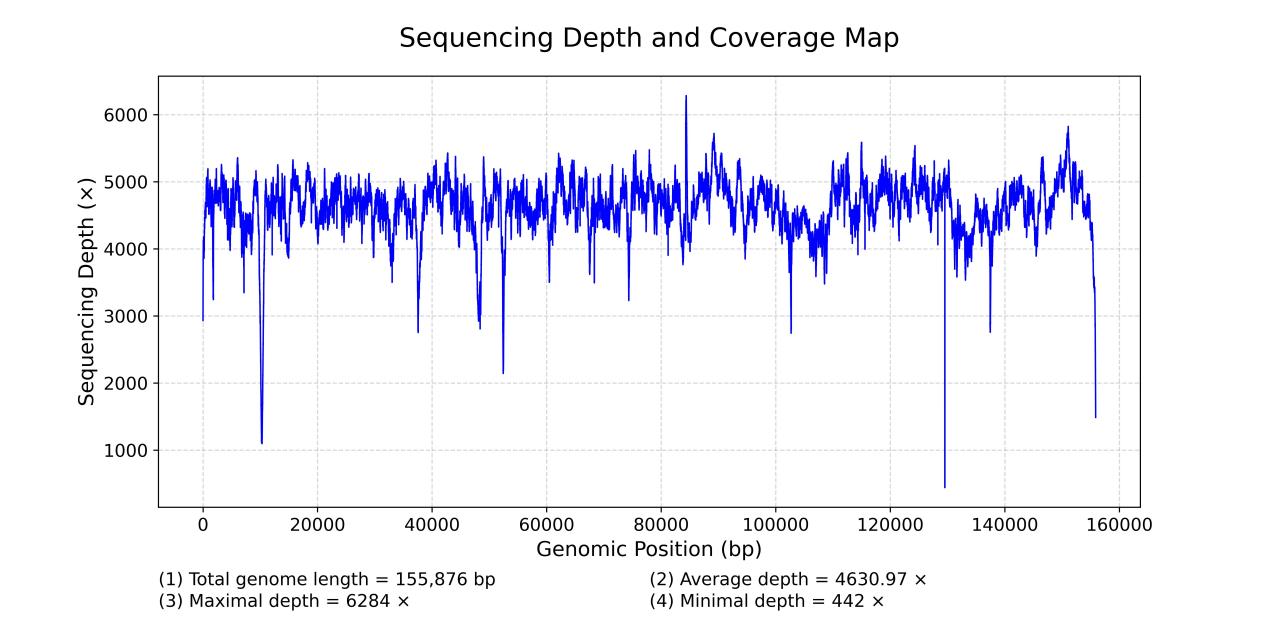


**Figure S1** Coverage depth distribution of the *S. uratensis*.


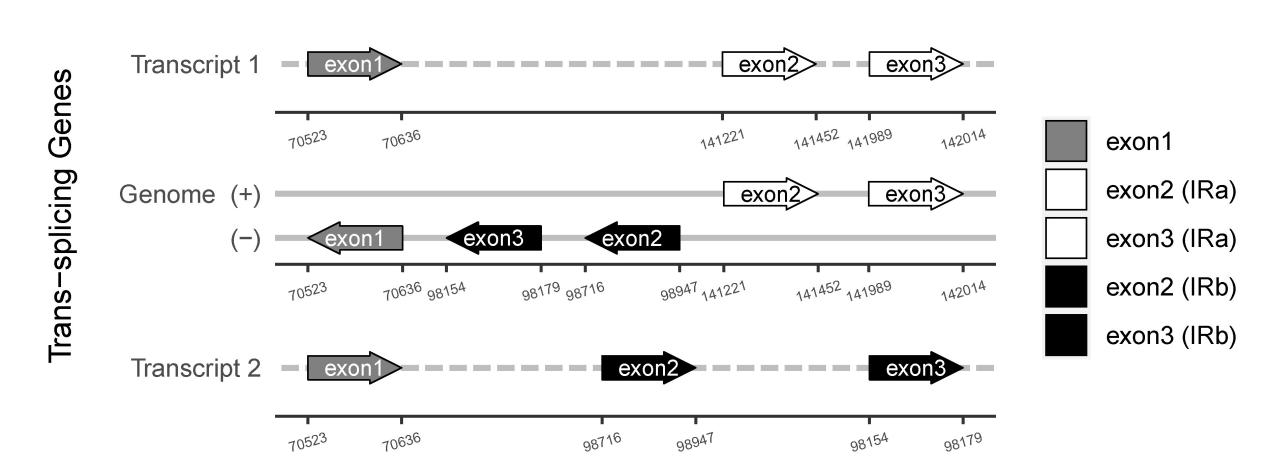


**Figure S2** Structure of trans-splicing genes in the *S. uratensis*.


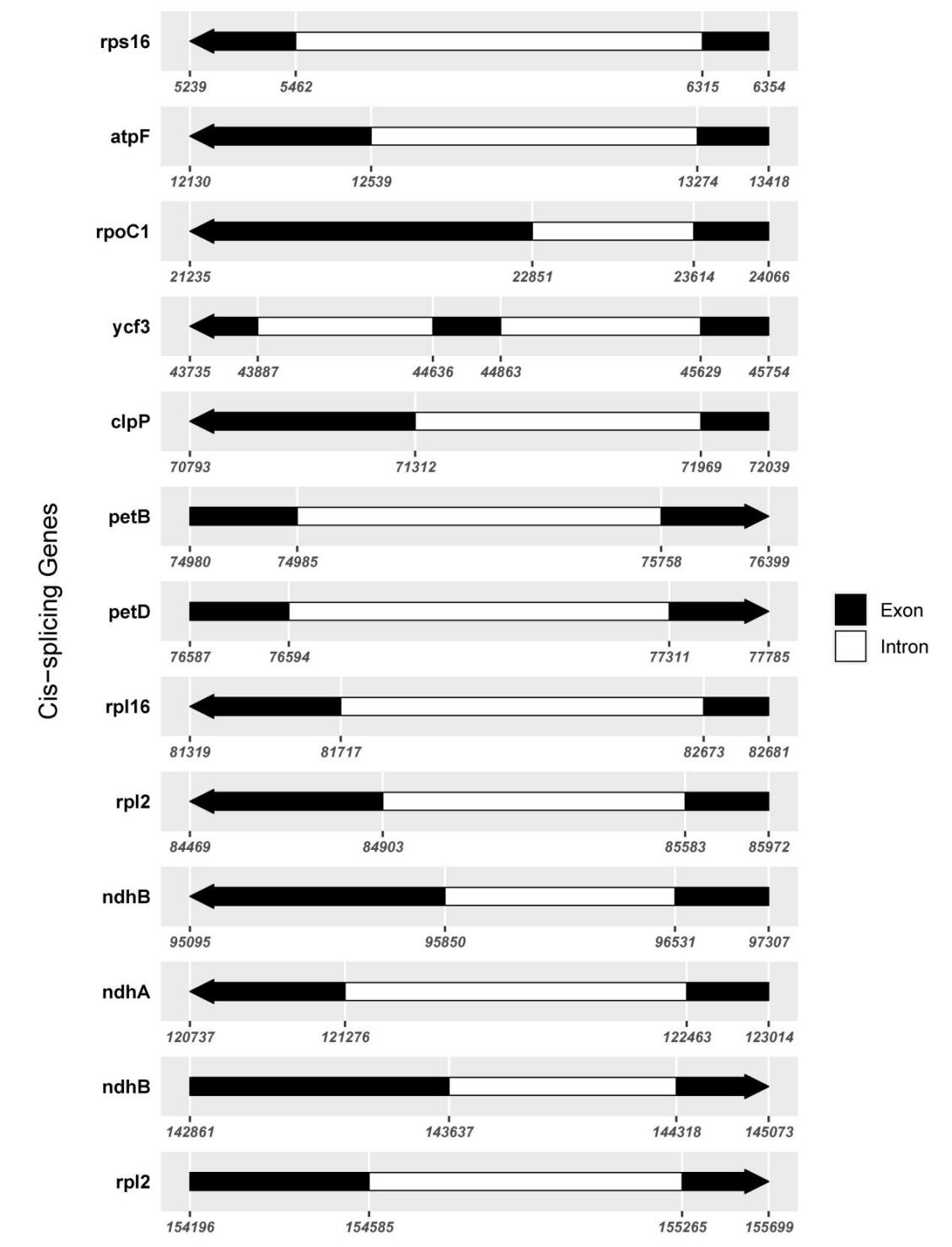


**Figure S3** Structure of Cis-splicing genes in the *S. uratensis*.
